# Rethinking LEO Mega-Constellation Routing to Provide Fast Internet Access Services

**DOI:** 10.3390/s23063207

**Published:** 2023-03-17

**Authors:** Zijian Yang, Feng Tian, Jifeng Jin, Huijie Liu

**Affiliations:** 1 Innovation Academy for Microsatellites of CAS, Shanghai 201203, China; 2 University of Chinese Academy of Sciences, Beijing 100049, China

**Keywords:** LEO mega-constellation, routing algorithm, space communications, satellite neworks, linear programming

## Abstract

In the realm of providing space-based internet access services, utilizing large-scale low Earth orbit (LEO) satellite networks have emerged as a promising solution for bridging the digital divide and connecting previously unconnected regions. The deployment of LEO satellites can augment terrestrial networks, with increased efficiency and reduced costs. However, as the size of LEO constellations continues to grow, the routing algorithm design of such networks faces numerous challenges. In this study, we present a novel routing algorithm, designated as Internet Fast Access Routing (IFAR), aimed at facilitating faster internet access for users. The algorithm consists of two main components. Firstly, we develop a formal model that calculates the minimum number of hops between any two satellites in the Walker-Delta constellation, along with the corresponding forwarding direction from source to destination. Then, a linear programming is formulated, to match each satellite to the visible satellite on the ground. Upon receipt of user data, each satellite then forwards the data only to the set of visible satellites that correspond to its own satellite. To validate the efficacy of IFAR, we conduct extensive simulation work, and the experimental results showcase the potential of IFAR to enhance the routing capabilities of LEO satellite networks and improve the overall quality of space-based internet access services.

## 1. Introduction

As the commercial deployment of fifth-generation (5G) communication systems advances globally, the research community has shifted its focus to the sixth-generation (6G) systems, which have the potential to usher in a paradigm shift in the field of communications [1]. One of the new frontiers of 6G development is the construction of large-scale low Earth orbit (LEO) satellite networks, which can effectively complement terrestrial networks and help to mitigate the disparities of the digital divide [2]. The LEO orbit, with its altitude ranging from 350 km to 2000 km, is centered on the Earth and offers a closer proximity to the planet compared to other orbits, resulting in reduced power requirements and latency, for satellite–ground communication.

However, the high velocity of LEO satellites, with respect to the ground, and the limitations of individual satellite beams, necessitate the deployment of mega-constellations, for continuous internet access in a target region. In recent years, numerous non-traditional technology companies have launched separate LEO mega-constellation programs, such as OneWeb, Amazon Kuiper, and SpaceX Starlink, involving the launch of thousands and tens of thousands of satellites, using reusable rockets [3]. With the rapid increase in the number of satellites, the overhead associated with route calculation and signaling transmission in the network becomes a critical challenge. Consequently, there is a pressing need to explore efficient and reliable routing algorithms.

The utilization of low Earth orbit (LEO) satellite networks to provide internet access services has been explored before, in the 1990s, however, most of these attempts did not succeed in fulfilling their intended purpose, e.g., GlobalStar [4] and Iridium [5]. The reasons behind their failure lay in their inability to compete with terrestrial telecommunications networks and insufficient funding to sustain the construction of such networks. The current focus on constructing LEO mega-constellations, aims to supplement terrestrial networks and provide internet access to remote regions, while also ensuring sustainable profitability.

In the literature, various current routing algorithms for LEO satellite networks have been proposed, aiming to improve the network throughput through multi-path transmission or load balancing [6,7,8]. These studies use satellites to build a core network in space, that transmits large amounts of data over long distances, through ISLs. However, the terrestrial core network is reasonably robust and resource-abundant now, and multi-hop transmission, through unstable ISLs, would bring additional repeat request overhead. Therefore, how to use the LEO mega-constellation as the access network to provide ultra-reliable and low-latency communication (URLLC) services remains an open issue, which inspired our work.

The main contributions of this paper include the following two aspects. First, we provide a theoretical model, to calculate the positions of satellites in the Walker constellation. This model is also used to derive a mathematical formula for the theoretical minimum number of hops between two satellites. The above calculation results are the basis of the satellite matching and forwarding strategy in IFAR algorithm. This algorithm operates in a distributed way and has low complexity, as only the routes between matching satellites need to be calculated.

The remainder of the paper is as follows. Section 2 presents the existing routing algorithms and the satellite model. The Internet Fast Access Routing (IFAR) algorithm is described in Section 3. The performance evaluation of the proposed algorithm is presented in Section 4, followed by the conclusions of the paper in Section 5.

## 2. Background

### 2.1. Related Work

The importance of routing in satellite networks has been mentioned by numerous papers in the context of the space information network [9,10]. Most algorithms were developed long ago and were driven by the needs of the time. For example, a routing algorithm for datagram traffic was proposed in [11], which can generate the minimum delay paths for each packet. This algorithm does not require frequent switching of paths to establish connections, as routes can be computed locally without the network topology information. However, in this paper, the packets are routes between logical nodes, mapped by the nearest satellite. When the constellation size becomes quite large, the mapping between the logical location and the satellite will become inaccurate, resulting in packets not being able to be transmitted to the correct destination.

In recent years, various routing algorithms based on LEO large-scale constellations, have been proposed [12,13,14,15]. In [12], Lai et al. measured large amounts of real-time communications (RTC) session data and found two critical causes of high latency in the current cloud-based wide-area RTC architecture: (i) additional latency introduced by underlying cross-domain protocols and suboptimal paths; (ii) insufficient cloud server deployments can also cause additional latency. To address the above issues, Lai et al. proposed an RTC framework called SPACERTC, which utilizes large-scale constellations, to help improve the latency performance of RTC session tasks. To be specific, SPACERTC first models the minimum RTC delay problem in spatial information networks and constructs a collaborative hybrid cloud network between the terrestrial cloud server and the constellation. Then, RTC streams are scheduled in this hybrid cloud network, to minimize the average RTC latency.

DRL-ER [13] is a routing algorithm based on deep reinforcement learning, which can balance link bandwidth resources and satellite battery resources. By modeling the energy consumption and acquisition in the large-scale satellite network into a Markov decision process (MDP), DRL-ER establishes the connection between link resources and routing decisions. Meanwhile, this algorithm trains the routing policies under different battery resources, through the experience replay strategy, to achieve better adaptability to delay bounds. To reduce the loss of data transmission failures in satellite networks, Zeng et al. designed RMPR [14], a multi-path routing algorithm with low-latency and failure tolerance. Compared with shortest path routing and backup dependent multi-path routing, RMPR transmits multiple copies over multiple independent shortest paths, in an optimal ratio, to ensure the reliability and timeliness of data. Grislain et al. were more interested in improving system throughput than reducing transmission latency, and proposed an innovative routing protocol in [15]. This protocol solves the indivisible multi-commodity flow problem in satellite networks, by a randomized rounding based heuristic algorithm, and optimizes the total amount of traffic across the constellation. Compared with the shortest path (SP) routing strategy, this protocol can significantly improve network throughput and alleviate network congestion.

Although there has been much research on routing algorithms for LEO mega-constellations, most of them still solve the end-to-end transmission problem, ignoring the most important application scenario of LEO constellations, which is to provide internet access services. We try to design a routing algorithm from a different angle, to meet the needs of fast internet access. Considering the large number of satellite nodes in mega-constellations, it is complicated and unnecessary to calculate the shortest path between all satellites. We only need to calculate the matches between satellites nodes and compute the routes between satellites in the matching set.

### 2.2. Satellite Network Model

This paper focuses on routing algorithms applied to a constellation called *Walker-Delta*. According to published regulatory filings, this configuration will be used in the mega-constellation designs proposed by many companies. In this constellation, the satellites will move in different circular orbital planes. Before mathematical modeling of the entire constellation, we first modeled the spatial position information of the satellite.

**Figure 1:**
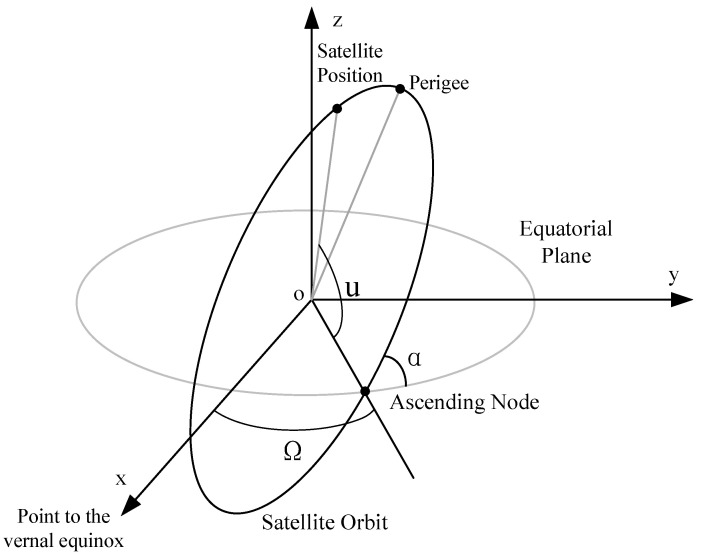
Schematic diagram of satellite orbit parameters.

The position of a satellite in space can be represented by classical orbital elements. In this paper, we are concerned about the *true anomaly, ν*, and the *longitude of the ascending node*, Ω. Since the satellite orbit is circular and the periapasis cannot be calculated, the *phase angle, u*, is used as a substitute parameter for the *true anomaly*, which represents the angle between the satellite and ascending node. The Ω at time *t*, is given by $\Omega =L_{0}-\omega_{e}\cdot t$, where $L_{0}$ is the initial longitude of the ascending node and $\omega_{e}$ is the rotational angular velocity of the Earth. The remaining elements, *inclination, α*, and *semi-major axis, a*, are constants of the constellation. The *semi-major axis, a*, is equal to the radius of the orbit, which is the sum of the orbit’s *altitude, h,* and the radius of the Earth. A schematic diagram of satellite orbit parameters is shown in Figure 1.

A Walker-Delta constellation described by α:PQ/P/F, contains *P* orbital planes. Each of these planes consist of *Q* equally spaced satellites, and the relative distance between adjacent plane satellites of this constellation is indicated by *F*. (i,j) is used to denoted the *j*-th satellite in orbital plane *i*, in the subsequent sections of this paper.

**Figure 2:**
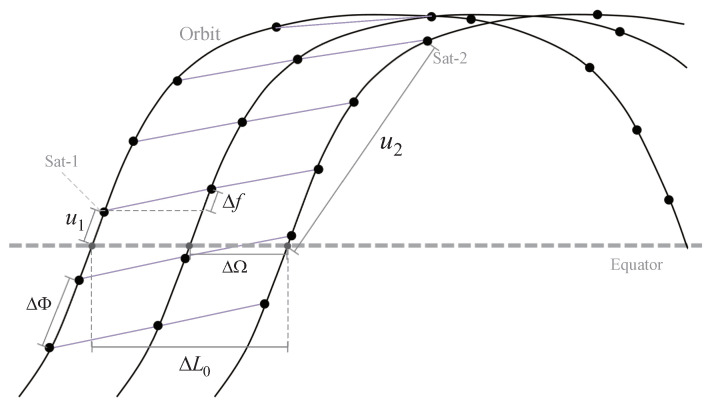
A partial constellation diagram marked by various modeling parameters.

The spacing between adjacent planes is specified by the right ascension difference of ascending nodes $\Delta \Omega =\frac{2\pi }{P}$, and the phase difference between adjacent satellites in an orbital plane $\Delta \Phi =\frac{2\pi }{Q}$, can be calculated by the number of satellites in each orbit. Finally, due to the existence of *the phase factor*, *F*, in the constellation parameters, the latitude of two horizontal neighbor satellites located in adjacent orbits are different, and the difference value is expressed as *phase offset*, $\Delta f=\frac{2\pi F}{PQ}$. A visualization of the above constellation parameters can be seen in Figure 2. Meanwhile, the satellite numbered (i,j) can be represented by these parameters as $\left(u=N\left(j\cdot \Delta \Phi +i\cdot \Delta f\right),L_{0}=N\left(i\cdot \Delta \Omega \right)\right)$, where N(x) is a normalized function ensuring that its result is in the interval [−π,π].

In the hypothesis of this paper, each satellite is equipped with four wireless terminals, to establish two intra-plane links and two inter-plane links with neighboring satellites. The notations for the preceding and subsequent satellites relative to the satellite (i,j) are denoted as (i,(j−1)modQ) and (i,(j+1)modQ), respectively. The neighbor to the left is denoted as (i−1,j) if i≠0, otherwise (P−1,(j−F)modQ). Homoplastically, the neighbor to the right is (i+1,j) if i≠P−1, otherwise (0,(j+F)modQ).

## 3. Our Proposed Algorithm

In this section, we present the Internet Fast Access Routing (IFAR) algorithm, which transmits user data to the ground core network as quickly as possible, without the need to calculate the optimal path of the whole network. The IFAR algorithm employs a matching strategy to associate landing satellites, which are responsible for establishing feeder links with Earth stations, with non-landing satellites. This association is performed in a manner that satisfies the link capacity constraints, while minimizing the number of hops required for data forwarding. Then, a linear programming, that can be solved in polynomial time, is used to match the landing satellite (satellite that establishes feeder links with Earth stations) and non-landing satellite, so that each non-landing satellite has corresponding landing satellites as the destination for data forwarding, under the premise of meeting the link capacity constraints. Finally, the optimal paths between the source and destination satellite sets are calculated by the forwarding strategy.

How to select the optimal access satellite for the user according to the satellite-user link quality and satellite available resources, is not considered in our algorithm. We work with the assumption that all the satellite–Earth topological relationships, including satellite–user links and satellite–Earth station (ES) links, are given in all the time intervals.

### 3.1. Minimum Hop Count

In the following, we present a model to determine the theoretical minimum number of hops between two satellites, based on the inter-satellite link (ISL) model put forth by Chen et al. [16]. We divide the hops between the two satellites into two parts, the intra-orbit hops and the inter-orbit hops.

The minimum number of inter-orbit hops, $N_{inter}$, of two satellites, can be calculated from the horizontal distance of the orbits of the satellites. Specifically, only the longitude difference of the ascending nodes is needed to calculate $N_{inter}$:

(1) $$\Delta L_{0}=\left(L_{0}^{1}-L_{0}^{2}\right)mod2\pi .$$

$\Delta L_{0}$ is the angular difference of longitude from the source orbit to the destination orbit in the east direction. Therefore, in the west direction, the angular difference of longitude can be expressed as $2\pi -\Delta L_{0}$. Since the longitude difference of adjacent satellites between orbital planes is ΔΩ, the minimum number of inter-orbit hops in the east and west directions are respectively given by:

(2) $$N_{inter}^{\to }=\frac{\Delta L_{0}}{\Delta \Omega }\;\;\;N_{inter}^{\leftarrow }=\frac{2\pi -\Delta L_{0}}{\Delta \Omega }.$$

When calculating the minimum number of intra-orbit hops connecting two satellites, it is necessary to consider the phase angle of the satellites. A hop to the neighbor satellite in an easterly direction between orbits increases the phase angle by Δf, while a hop to the subsequent satellite in the orbit increases the phase angle by ΔΦ. When only the hops to the adjacent satellites in the right orbit and the hops to subsequent satellites are taken into account, the following formula is true:

(3) $$u_{2}=u_{1}+\Delta \overset{\leftarrow }{u}+\left(N_{inter}^{\to }\cdot \Delta f\right)\;\;\;\Delta \vec{u}=\left(N_{intra}^{↗}\cdot \Delta \Phi \right).$$

where $N_{intra}$ is the minimum number of intra-orbit hops we need to calculate. The phase angle difference caused by intra-orbit hops in one direction, $\Delta \vec{u}$, can be obtained by converting the above formula:

(4) $$\Delta \vec{u}=u_{2}-N_{inter}^{\to }\cdot \Delta f-u_{1}.$$

Similarly, the phase angle difference in the other direction, $\Delta \overset{\leftarrow }{u}$, can be obtained by the following formula:

(5) $$\Delta \overset{\leftarrow }{u}=u_{2}+N_{inter}^{\leftarrow }\cdot \Delta f-u_{1}.$$

Finally, the minimum number of intra-orbit hops, $N_{intra}$, can be obtained by the phase difference between adjacent satellites in orbit and the phase difference between the source and destination satellites in orbit:

(6) $$\begin{array}{cc} N_{intra}^{↖}=\frac{\Delta \overset{\leftarrow }{u}}{\Delta \Phi } & N_{intra}^{↗}=\frac{\Delta \vec{u}}{\Delta \Phi }\\ N_{intra}^{↙}=\frac{2\pi -\Delta \overset{\leftarrow }{u}}{\Delta \Phi } & N_{intra}^{↘}=\frac{2\pi -\Delta \vec{u}}{\Delta \Phi }. \end{array}$$

Therefore, the theoretical minimum number of hops between the two satellites, *N*, is given by the minimum in the following combination:

(7) $$\min\{N_{inter}^{\leftarrow }+N_{intra}^{↖},N_{inter}^{\leftarrow }+N_{intra}^{↗},N_{inter}^{\to }+N_{intra}^{↙},N_{inter}^{\to }+N_{intra}^{↘}\}.$$

Meanwhile, the minimum value in the forwarding direction combination above represents the reference forwarding direction of the minimum hops path.

### 3.2. Satellite Set Matching Strategy

In the traditional routing algorithm, each satellite needs to calculate the optimal path to the other satellites in the entire satellite network and store the corresponding forwarding table. However, in the routing algorithm proposed in this paper, each satellite only needs to calculate the route to the landing satellite in this satellite set. In this section, we will introduce the satellite matching strategy.

**Figure 3:**
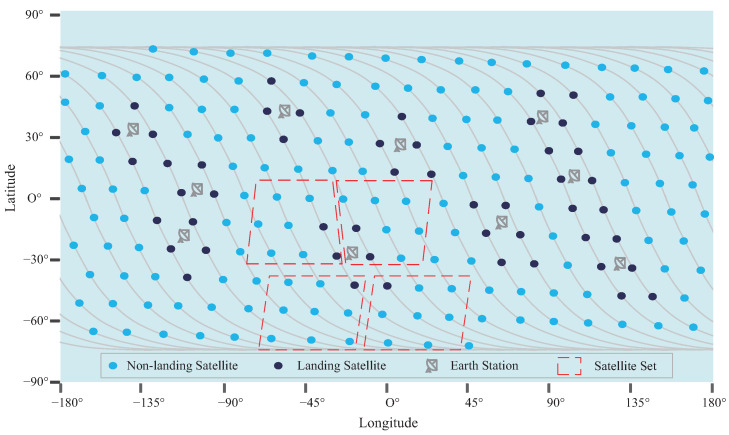
A diagram of satellite sets in a Walker constellation.

The purpose of the matching strategy is to assign one or more landing satellites to each satellite, and the landing satellite establishes a connection with the ground station through a feeder link, at the current time. When the satellite receives an internet access request from a ground-based user, it sends the data directly to a matching landing satellite. Users will be connected to the ground core network through the feeder link of the landing satellite. The purpose of this approach is twofold: first, minimize the hop number of satellite network packets to reduce the internet access delay and avoid packet loss caused by unstable ISLs; second, greatly reduce the complexity of the routing algorithm. Each set of satellites includes several non-landing satellites and landing satellites, as shown in Figure 3. Note that, for the sake of brevity, only the satellites in ascending orbit are shown in this figure.

The following linear programming is used in this paper to match satellites. First, we divide all satellites into the landing satellite set D, and the non-landing satellite set S. Then, in order to improve the comprehensive user experience of the whole network, our optimization goal is to minimize the total hops from each satellite to its matching satellite, as shown below:

(8) $$Min:\sum_{s\in S}\sum_{d\in D}W_{s,d}\cdot N_{s,d}.$$

where, $W_{s,d}$ are the separate flow ratio of source satellite to destination satellite, defined as the ratio of the traffic transmitted to the destination satellite to the total traffic received by the source satellite. $N_{s,d}$ are the theoretical minimum hops from the source satellite to the destination satellite, which are obtained in the previous subsection.

Meanwhile, considering the user’s data transmission requirements, and the capacity of the feeder link to establish the connection between the satellite network and the ground, there are the following constraints:

(9) $$Subject\;to:\sum_{d\in D}W_{s,d}=1\;\;\;\forall s\in S$$

(10) $$\;\;\;\;\;\sum_{s\in S}T_{s}\cdot W_{s,d}\leq C_{d}\;\;\forall d\in D.$$

where $T_{s}$ represents the data traffic received by the source satellite *s*, and $C_{d}$ represents the feeder link capacity of satellite *d*. Formula (9) defines the constraint of user transmission demand, to ensure that all user data can be transmitted to the ground core network. Formula (10) defines the capacity constraint of feeder links, that is, the data rate transmitted on a feeder link cannot exceed its capacity.

### 3.3. Forwarding Strategy

When the satellite set is determined, each non-landing satellite in the set will forward data to each landing satellite in a certain proportion. Meanwhile, two reference directions for data forwarding have been given by the theoretical minimum hops between satellites. Each satellite flips a coin, to randomly select one of two reference directions as the forwarding direction of the next hop. In addition, in order to limit the forwarding path to a rectangular range of a spherical surface, with the source satellite and the destination satellite as two endpoints, the rest of the path is forced to select the horizontal hops to complete after the packet has passed through all the vertical hops.

By adopting such a probabilistic forwarding mode, the forwarding path of data packets can have enough randomness so that the ISL’s capacity resources can be fully utilized, and the load balancing of network resources can be realized without the need for centralized routing calculation.

## 4. Evaluation

In this section, we provide the performance evaluation results of the software simulation, to evaluate the performance of our proposed algorithm. We implemented the constellation model and deployed the IFAR algorithm by STK (Satellite Tool Kit) and OPNET Modeler. OPNET Modeler is a network simulation software, which promotes the simulation process by triggering various discrete events. All simulations were run on a Windows machine, with a 2.9 Ghz Inter Core i7-10700 CPU and 16 GB of RAM. We take the average packet loss rate and average transmission delay under different loads as performance metrics. To verify the effectiveness of the IFAR algorithm, we compared it with OSPF (Open Shortest Path First), a routing algorithm commonly used in LANs (local area networks).

### 4.1. Experiment Setting

**Figure 4:**
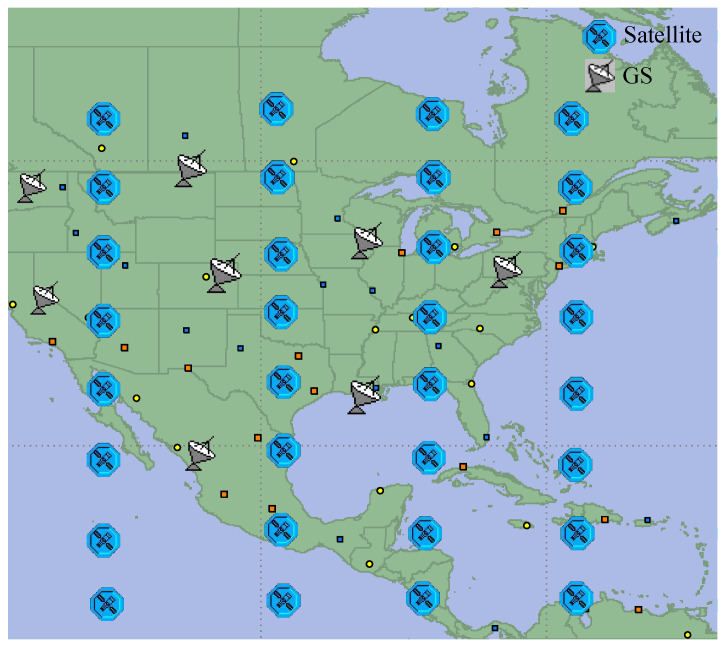
Constellation diagram in the simulation software.

We referenced the constellation parameters of first shell in Starlink’s initial deployment and built a Walker-Delta constellation to evaluate the algorithm’s performance. The specific parameters of the constellation are: 53.0:500/20/11, and the orbital altitude is 550 km. The constellation diagram, in the simulation software OPNET, is shown in Figure 4. Each satellite node moves according to the defined orbital parameters, and when it runs within the visible range of the ground station, it can establish a feed link with the ground station and become a landing satellite. At the same time, in order to simulate the traffic differences due to geographical factors, we divide the world into several square grids, each grid is assigned a traffic weight, based on population density and development level. The traffic generated by the traffic model, multiplied by the weight of the region, is the real traffic of users accessing the satellite network in this region.

Each satellite can use a phased array antenna to build a satellite-user link and the internet service request rate of users is in a Gaussian distribution $N(\mu ,\sigma^{2})$, where μ represents the mean and $\sigma^{2}$ represents the variance of this distribution. In the simulation, we model different network load conditions by changing the mean, μ. Each satellite can establish ISLs with two satellites in the same orbit and two satellites in the adjacent orbit, through the wireless transceiver terminals. Due to all kinds of electromagnetic interference in space, ISLs are unstable. In this paper, based on our experience in engineering testing, we assume that the packet loss rate of a single ISL is 0.5%.

**Figure 5:**
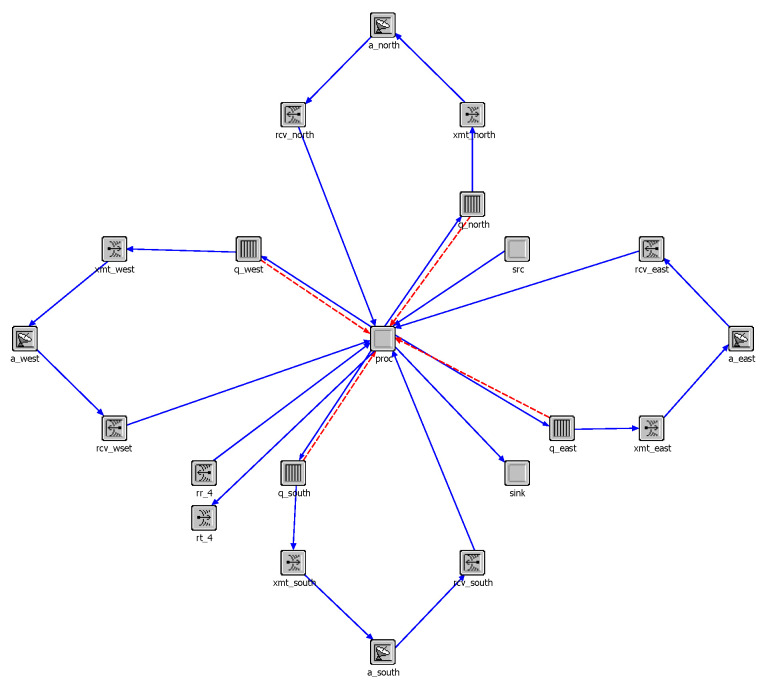
The model of satellite nodes in the simulation software.

In the simulation environment, each satellite node in Figure 4 is composed of the satellite node model shown in Figure 5. In the satellite node model, each satellite has four wireless transceiver terminals, to establish ISLs with neighboring satellites. Each transmitting terminal is equipped with a cache queue, for temporarily storing data packets that are not sent out in time. The “src” module is used to generate user data received by each satellite, the “sink” module is used to delete useless data packets, and the “proc” module is mainly responsible for implementing the network layer protocol and the algorithm proposed in this paper. Solid lines with arrows between different modules indicate the direction of data flow between these modules, and dotted lines with arrows indicate the monitoring and acquisition of some parameters in these modules.

It should be noted that we assume that the terrestrial network is very robust, without packet loss and congestion. In this way, we can better show the performance of the satellite network in data transmission.

### 4.2. Results and Analysis

(1) Average Internet Access Latency:We conducted simulations to evaluate the delay performance of the IFAR algorithm on a large-scale LEO constellation of 500 satellites, and compared it with the OSPF algorithm. To quantify the network’s performance, we defined the network relative load as the ratio of all user requests received by the satellite network to the total bandwidth of the feeder link.

**Figure 6:**
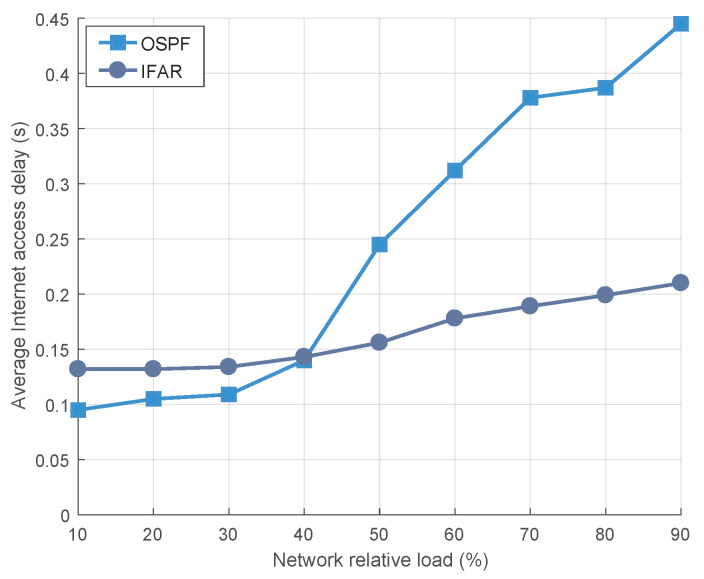
The average internet access latency of users under different network loads.

We conducted experiments under varying network relative loads, as illustrated in Figure 6. The average internet access delay is defined as the average delay between the packet sent from the user terminal to the satellite network and successfully downloaded from the satellite network to the terrestrial server. Our findings revealed that when the network load is light, the OSPF algorithm outperforms the IFAR algorithm, in terms of delay performance. This can be attributed to the fact that the IFAR algorithm rapidly transmits data to the ground station and subsequently accesses the server through ground optical fiber, while the OSPF protocol first forwards data packets to the ground station closest to the server through the ISLs, before accessing the server. As a result, data forwarded by the OSPF algorithm travels a longer distance in space. Because the speed of light in space is 40% faster than the speed of light in optical fiber, packets can travel faster on satellite networks than on the ground, when there is no congestion.

As the network load increases, the experiment using the OSPF algorithm results in a considerable number of data packets being forwarded on the satellite network. This causes the bandwidth resources of the ISLs to gradually deplete, resulting in packets being unable to be forwarded promptly in the cache. Network congestion ensues, which in turn increases the queuing delay of packets, consequently impacting the internet access delay of users. In contrast, in the simulation utilizing the IFAR algorithm, data will be quickly downloaded from the matching landing satellites to the ground network, and then the abundant and robust optical fiber will be used for data transmission. Consequently, a large number of packets are prevented from traveling long distances through the ISLs, thereby effectively mitigating congestion in the satellite network.

**Figure 7:**
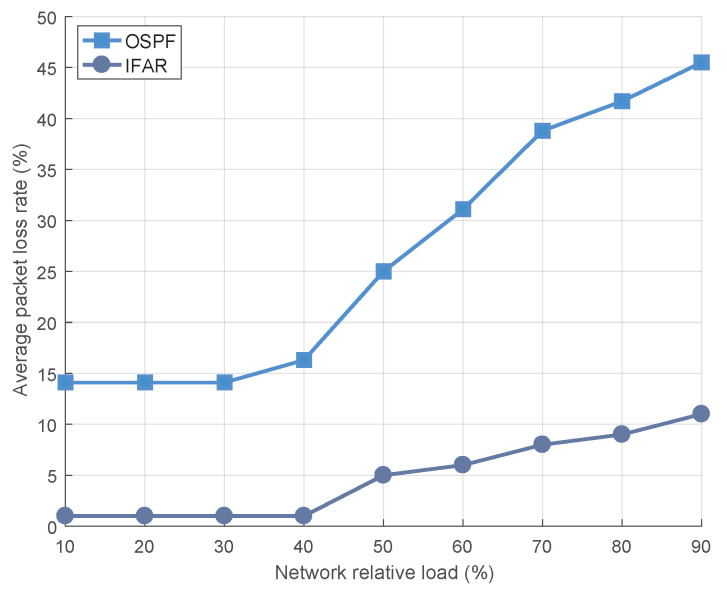
The average packet loss rate of users under different network loads.

(2) Average Packet Loss Rate: As the ISL is not stable enough, it will often fail and cause packet loss. Therefore, a large number of packets forwarded by ISLs may not only cause congestion, but also increase the packet loss rate, as shown in Figure 7. The average packet loss rate is defined as, the ratio of packet loss caused by unstable inter-satellite links to the total number of packets received by the satellite network.

When the network load is low, the OSPF algorithm resorts to forwarding packets on the ISL through multiple hops, thereby compounding the packet loss rate and resulting in a high average network packet loss rate. In contrast, the IFAR algorithm relies on the stable and resource-rich ground network to forward more packets, effectively circumventing packet loss caused by the instability of ISLs. As the network load increases, the satellite network becomes increasingly congested, and the packet loss rate of both algorithms starts to rise. This is because the OSPF algorithm discards a substantial amount of packets, that either exceed their lifetime limits or the cache capacity is insufficient. The IAFR algorithm can only transmit data between matching satellites, avoiding long-distance transmission by ISLs, and meeting the needs of users for fast access to the internet.

## 5. Conclusions

In this paper, we have presented a novel routing algorithm for achieving fast internet access, thereby enabling users to enjoy a superior online experience. As the ISL is known to be less stable compared to ground network equipment, it is essential to avoid multi-hop transmissions via the ISL. To address this issue, we first established a formal model and calculated the theoretical minimum hops between two given satellites. Then, we proposed a linear programming to match the satellites between the landing satellite and the non-landing satellite. Finally, only the routes between the satellites in the matching set need to be calculated. In the simulation section, we leveraged the OPNET modeler to construct a large-scale LEO satellite network by referring to the Starlink network configuration, and used it to evaluate the performance of the proposed algorithm. The simulation results indicate that the IFAR algorithm can significantly enhance the internet access speed and substantially decrease the network packet loss rate.

## Data Availability

The data presented in this study are available on request from the corresponding author.
